# Helium Hand – High Pressure Injection Injury

**DOI:** 10.5334/jbsr.3139

**Published:** 2023-05-12

**Authors:** Brandon Funk, Francis Flaherty, Edward Gillis

**Affiliations:** 1 Norwalk Hospital, US

**Keywords:** high pressure injection, helium, hand injury, subcutaneous emphysema, compartment syndrome

## Abstract

**Teaching Point:** High-pressure injection injuries are clinically significant injuries which may be underappreciated on initial physical exam and which require a high index of suspicion and early clinical intervention to avoid negative outcomes.

## Case History

**Figure 1:**
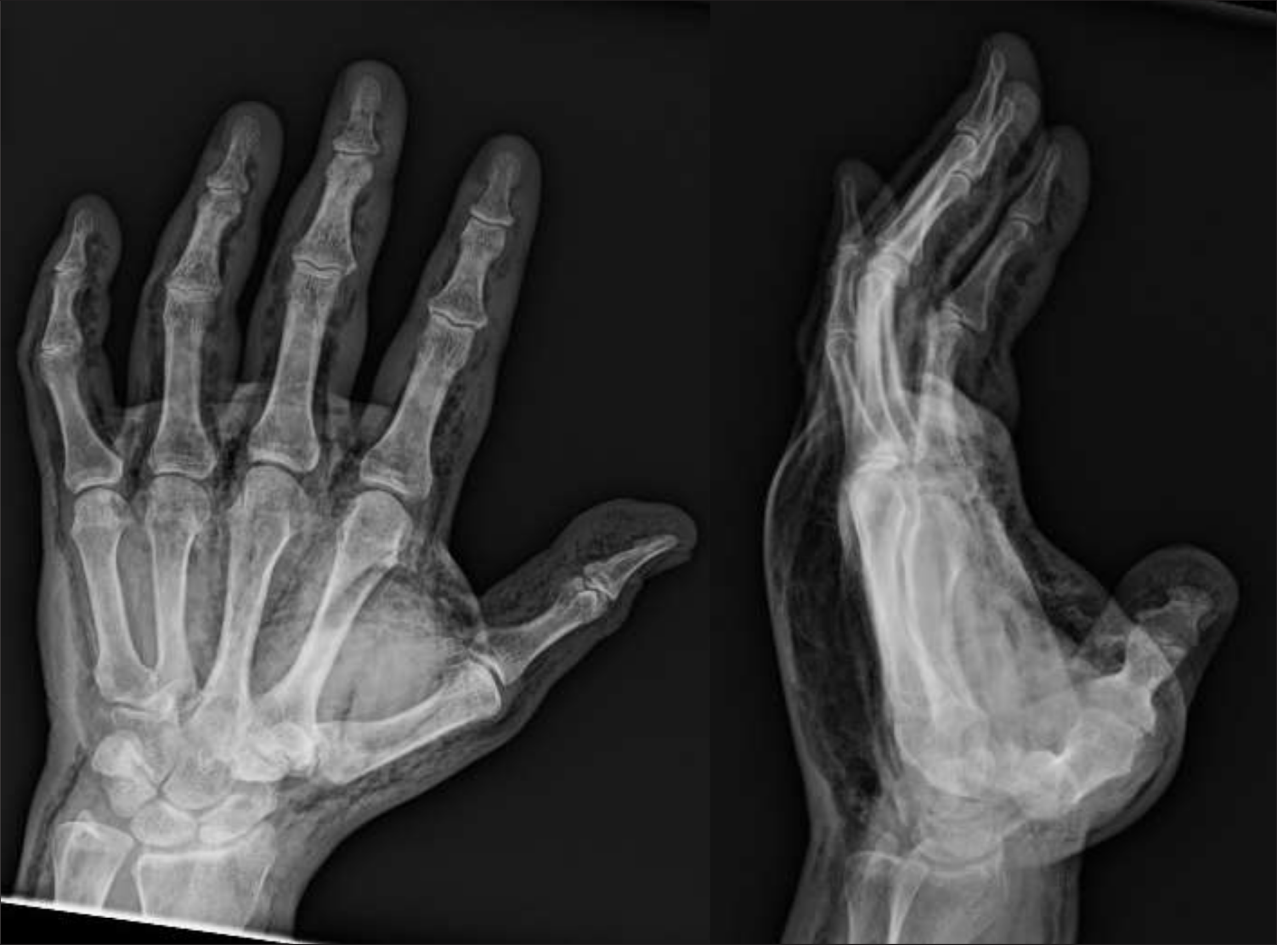


A 46-year-old female presented to the ER with diffuse swelling of the left hand after attempting to fill a balloon from a helium tank. The patient’s distal thumb exhibited a punctate entry wound, pale tense skin, and absent capillary refill. Radiographs showed extensive diffuse subcutaneous emphysema consistent with helium insufflation (Figure 1) injury. Due to suspected compartment syndrome, the patient underwent a decompressive fasciotomy of the thumb with subsequent full recovery.

## Comments

High-pressure injection injuries are caused by high-pressure delivery devices such as paint guns or pressurized gas tanks [1]. Injury severity may be underappreciated on initial assessment given the tiny entry wound [1]. Emergent treatment, including immediate surgical debridement, is frequently needed to avoid digit loss or long-term complication. The need for debridement depends on the volume and nature of the material injected (gas, paint, grease, etc.), which will determine the risk of compartment syndrome and the degree of local adverse tissue damage [1].
